# Baseline Neurotracker performance does not correlate to overall season sport performance in university varsity athletes

**DOI:** 10.3389/fspor.2025.1608463

**Published:** 2025-10-29

**Authors:** Jean-Michel Acquin, Elizabeth Giguère-Lemieux, Alexandre Deschamps, Julien Glaude-Roy, Jean-François Brunelle, Philippe Fait, Laurie-Ann Corbin-Berrigan

**Affiliations:** Department of Human Kinetics, Université du Québec à Trois-Rivières, Trois-Rivières, QC, Canada

**Keywords:** sports performance, 3D-MOT, Neurotracker, ice hockey, volleyball, perceptual-cognitive abilities

## Abstract

**Introduction:**

This study examined the relationship between preseason Neurotracker performance and sports performance in university varsity male ice hockey and female volleyball athletes.

**Methods:**

Neurotracker, a 3D multiple object tracking task, was used to assess perceptual-cognitive abilities. The association between these abilities and sport statistics was assessed through correlations.

**Results:**

48 university varsity athletes completed a Neurotracker baseline assessment every preseason between 2019 and 2022, and competed in male ice hockey or female volleyball regular seasons. No significant correlations were found for all sports statistics in female volleyball athletes and male ice hockey except for total penalty minutes (r =  -,40, p < 0,05) and penalty minutes per game (r =  -,39, < 0,05) in male ice hockey.

**Discussion:**

The findings indicate that Neurotracker performance is not associated with sports performance. Further research would be interesting to explore more specific to perceptual-cognitive skills sports statistics in this context.

## 1 Introduction

Neurotracker is a 3D multiple object tracking (3D-MOT) task developed to train perceptual-cognitive abilities and improve sports performance (1) by targeting attention (sustained and divided), visual tracking, decision-making skills, executive functioning and working memory (2, 3). Perceptual-cognitive abilities consist of processing prompt visual information, to make the best decision and execute actions that integrate several stimuli. In the context of sports, the Neurotracker aims to train attention (sustained, divided), visual tracking, decision-making skills, executive functionning and working memory (2). These skills are essential for athletes to meet the demands of high-level sports (1, 4). In fact, studies have shown that high-level athletes have better cognitive functions than low-level athletes (5–7) and improving perceptual-cognitive abilities could help athletes reach higher levels in their respective sport (8) by enhancing sport awareness and improving sport-specific decision making on the field, especially in team settings (2). Some studies have shown that Neurotracker training not only enhances perceptual-cognitive skills, but also exhibits potential improvements in working memory, sustained attention, and processing speed or inhibition across diverse populations (1). Moreover, the ability to track multiple objects in a 3D environment is an asset that can be transferred to sports practice, thereby enhancing performance (9, 10).

Previous studies support that Neurotracker could help identify markers of performance or talent in different contexts (daily activities and professional activities) (11, 12). Indeed, research findings have established a correlation between Neurotracker performances and driving skills, with this tool emerging as an effective predictor of heightened crash risk in older drivers (11, 12). A study also revealed that Neurotracker performance could predict laparoscopic surgical skills, with residents with higher Neurotracker scores exhibiting shorter surgery time and less movement in the surgical arms, resulting in more precise surgeries (13). Furthermore, Jarvis et al. (14) showed that Neurotracker could help in air traffic controllers selection (14). These findings suggest that candidates with higher Neurotracker scores had better performance in four of five air traffic control tasks (conflict detection, false alarm response, response time for aircraft acceptance and hand-off) (14).

In sports, Neurotracker holds promise as a potential tool for talent identification and performance correlations with applications in various sport contexts, including university varsity and professional sports (10, 15–17). Indeed, studies suggest that expert athletes perform better in multiple object tracking compared to amateur athletes, and that perceptual-cognitive skills can be enhanced in various athletic populations (2). This underscores the idea that Neurotracker may serve as a discerning measure of performance in athletes (8, 18). For example, Mangine et al. (15) demonstrated that Neurotracker performance seems to be related to basketball performance in professional athletes (15). Athletes exhibiting higher Neurotracker performance a week before the National Basketball Association (NBA) regular season presented better basketball-specific performances throughout the season as opposed to those with lower baseline scores. Given that Neurotracker performance across various populations is enhanced by repetitive exposure to the training paradigm, if the Neurotracker was found to correlate to sport performance, one could argue that said performance may be enhanced through repetitive exposure to the task (18). Hence, assessing the role of Neurotracker performance in athletes could be beneficial in both talent identification, but mostly for sport-performance assessment and potential training of athletes across various sports. Therefore, the aim of this study was to examine the correlation between baseline preseason Neurotracker performance and sports performance among university varsity male and female athletes. We hypothesize that higher Neurotracker performance will correlate with sports statistics, more specifically pertaining to decision making.

## 2 Methods

### 2.1 Participants

A convenience sample of university varsity athletes from the Université of Québec in Trois-Rivières (UQTR), engaging in sports for which player statistics are publicly available on USPORTS or Réseau du sport étudiant du Québec (RSEQ) websites. Participants were recruited prior to the beginning of the sporting seasons between the years 2019 and 2022, inclusively (Table 1). Participants were included if they were playing one of the following university varsity sports: male ice hockey or female volleyball and were fluent in both spoken and written French or English. Participants were excluded if they had an uncorrected vision problem, major neurological or orthopedic disorder limiting the completion of the assessment, and if they reported a recent prior concussion (less than three months at the time of the study). The project was approved by the UQTR's research ethics committee (CER-14-205-07.17) and written consent was obtained from all participants.

**Table 1:** Demographic characteristics of participants.

Participants (*n*=)	Age (years)	Sports	Sport experience (years)	Positions (*n*=)
Female: 14	22.57 ± 2.95	Volleyball	11.86 ± 2.14	Hitter: 6 Libero: 2 Middle blocker: 4 Setter: 2
Male: 34	23.29 ± 1.57	Hockey	18.03 ± 1.77	Defenseman: 11 Forward: 23

### 2.2 Neurotracker

**Figure 1:**
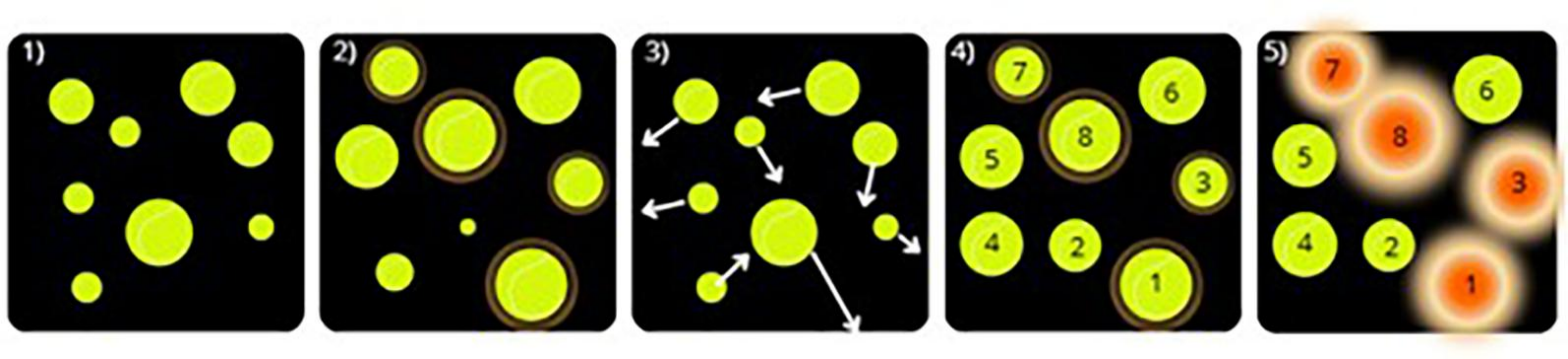
Visual representation of the Neurotracker's steps (1–5).

Every preseason between 2019 and 2022 (months of August and September), prior to regular season games, participants completed a baseline Neurotracker assessment. During the assessment, participants were seated in a dark room wearing active 3D glasses, 1,5 meters from the interface projection. Baseline Neurotracker performance was established with the CORE program, using four targets, which is widely reported in the literature (1). One session of Neurotracker CORE program comprises three blocks of 20 trials. One trial involves the following five steps: (1) eight yellow balls are randomly distributed in the 3D interface; (2) the color of the four targets to be tracked during the trial change to orange for two seconds; (3) the four targets return to their initial color (yellow) and the eight balls start moving in the interface for eight seconds; (4) the balls stop moving and numbers (1–8) appear on each of them and the participant have to verbally or manually (on a keypad) identify the four targets that were previously establish at step 2; (5) the trial result is shown to the participant and the next trial starts immediately afterward, following the same steps, with a different speed determined by the success/failure of the previous trial (Figure 1). Speed is increased by the Neurotracker CORE program after a successful trial, while speed is reduced after an unsuccessful trial. Consistent with research studies previously published with the Neurotracker, after each block of 20 trials, a speed threshold is established, representing the maximal speed, in meter per second (m/s), at which participants can accurately track 4 moving targets, and after the three blocks, a mean speed threshold is obtained, which is referred to as baseline Neurotracker performance (1). For the purpose of this study, if participants were tested over multiple years (i.e., engaged in university varsity sports for more than one year during the study period), only the most recent baseline performance score was retained for analysis.

### 2.3 Sports statistics

The selected ice hockey outcome measures were number of regular season games played, regular season total goals, regular season total assists, regular season total points (sum of goals and assists), regular season total penalty minutes, goals per game, assists per game, points per game and penalty minutes per game for each season between 2019 and 2022. Forwards and defensemen were grouped separately due to the playing style and requirements between the positions.

Volleyball outcome measures gathered for this study were sets played, hitting percentage (total kills minus total errors divided by total attempts), assists per set, serve percentage, reception percentage (serve receptions—serve reception errors divided by total serve receptions), digs per set (a dig is recorded when a player successfully pass the ball that has been attacked by the other team), blocks per set (blocks leading directly to a point) and points per set (kills + service aces + block solos + 0.5/block assists divided by sets played) for the 2022 regular season.

Data were respectively collected on USPORTS and RSEQ official websites and are publicly available, also justifying the choice of selected outcome measures. Coherently to Neurotracker performance, for the purpose of this study, if participants were tested over multiple years (i.e., engaged university varsity sports for more than one year during the study period), only the most recent sports statistics were used.

### 2.4 Statistical analysis

Descriptive data were used to present participants' characteristics. Due to the non-normality of data, non-parametric statistics were opted for. Pearson's correlations were used to establish statistical relationships between Neurotracker performance, reported as speed thresholds, and sports performance, as reported by overall sport statistics, and significance level was set a *p* < 0.05. In male ice hockey players, the relationship between Neurotracker and sport performance was also studied based on player position (forward vs. defense). Such analyses were not possible for female volleyball players due to the relatively small sample size and variation amongst player positions. Statistical analyses were conducted on IBM SPSS Version 29.0.

## 3 Results

48 university varsity athletes (70,8% male, mean age: 23.08 ± 2.06 years) were included in the study. Demographics characteristics of participants can be found in Table 1. Descriptive statistics for hockey and volleyball are presented in Table 2. The correlations between Neurotracker performance and each hockey statistic are presented in Table 3. Significant correlation with Neurotracker average speed threshold were also for the number of games played (*r* *=* -.381, *p* *<* 0.05) and total penalty minutes (*r* *=* -.375, *p* *<* 0.05). Correlations were also run between Neurotracker performance and hockey statistics for the forwards and defensemen groups. A significant correlation was found in the forwards group (Table 4) between Neurotracker average speed threshold and number of games played (*r* *=* -.443, *p* *<* 0.05).

**Table 2:** Descriptive statistics of hockey and volleyball university varsity athletes.

Male ice hockey	Mean ± Std. deviation (x¯ ± SD)	N	Female volleyball	Mean ± Std. deviation (x¯ ± SD)	N
Hockey NT Preseason Average Speed Threshold (m/s)	1.44 ± 0.31	34	Volleyball NT Preseason Average Speed Threshold (m/s)	1.14 ± 0,25	14
GP	19.24 ± 7.48	34	SP	49.29 ± 20.55	14
G	4.00 ± 4.38	34	Hitting %	0.04 ± 0.18	14
A	7.15 ± 5.85	34	A/Set	0.76 ± 2.08	14
Pts	11.15 ± 8.66	34	Serve %	0.81 ± 0.24	14
PM	13.62 ± 10.87	34	Recept %	0.78 ± 0.29	14
G/Game	0.21 ± 0.26	34	Dig/S	1.20 ± 0.77	14
A/Game	0.35 ± 0.24	34	Blk/S	0.16 ± 0.20	14
Pts/Game	0.56 ± 0.39	34	Pts/S	1.15 ± 1.14	14
PM/Game	0.66 ± 0.48	34			

A, total assists; A/Game, assist per game; A/Set, assists per set; Blk/S, blocks per set; Dig/S, digs per set; G, total goals; G/Game, goal per game; GP, games played; N, number; NT, neurotracker; m/s, meter per second; Hitting %, hitting percentage; Recept %, reception percentage; Serve %, serve percentage; PM, total penalty minutes; PM/Game, penalty minute per game; Pts, total points; Pts/Game, point per game; Pts/S, points per set; SP, sets played.

**Table 3:** Correlations between Neurotracker performance and hockey statistics.

		GP	G	A	Pts	PM	G/game	A/game	Pts/game	PM/game
NT Preseason Average Speed Threshold (m/s)	*r*	**-**.38^*^	.07	.07	.09	**-**.38^*^	.24	.26	.32	-.28
	p	.03	.69	.67	.63	.03	.18	.14	.07	.11
	Forward									
	*r*	**-**.44^*^	-.05	-.10	-.09	-.29	.18	.14	.20	-.14
	p	.03	.81	.64	.69	.18	.41	.53	.37	.53
	Defense									
	*r*	-.12	-.18	.43	.39	-.25	-.16	.52	.51	-.15
	p	.72	.59	.18	.24	.45	.64	.10	.11	.66

Values in bold represent where significance level was reached. A, total assists; A/Game, assist per game; G, total goals; G/Game, goal per game; GP, games played; NT, neurotracker; m/s, meter per second; PM, total penalty minutes; PM/Game, penalty minute per game; Pts, total points; Pts/Game, point per game; Sig., significance level. *p < 0.05.

**Table 4:** Correlations between Neurotracker performance and volleyball statistics.

		SP	Hitting %	A/Set	Serve %	Recept %	Dig/S	Blk/S	Pts/S
NT preseason average speed threshold (m/s)	*r*	.31	-.09	.12	.17	.04	.20	.28	.37
	p	.27	.77	.69	.57	.88	.50	.33	.19

A/Set: Assists per set, Blk/S: Blocks per set, Dig/S: Digs per set, NT: Neurotracker, m/s: meter per second, Hitting %: Hitting percentage, Recept %: Reception percentage, Serve %: Serve percentage, Pts/S: Points per set, Sig.: Significance level, SP: Sets played.

No significant correlations were found between Neurotracker performance and hockey statistics for the defensemen (Table 3) and volleyball athletes (Table 4).

## 4 Discussion

The aim of our study was to examine the relationship between baseline Neurotracker performance and overall season sports performance in university varsity male ice hockey and female volleyball athletes. We hypothesized that Neurotracker performance would correlate to sport performance as represented by player statistics. When looking at Neurotracker average speed thresholds, we found no significant positive correlations for all sports statistics in male ice hockey and in female volleyball athletes,but found significant negative correlations for game played and penalty minutes in male ice hockey.

A weak but statistically significant negative correlation was found between game played and Neurotracker performance. This relationship should be approached with caution as it is not supported by existing theory or prior research findings. The amount of game played by hockey athletes is highly dependent of team strategy and injuries preventing play (19, 20). In university level ice hockey, knee injuries and concussion account for most reported injuries (21). While is it suggested that most injury at this level of play occur as a result of player-to-player contact, the rate of overuse injuries should not be disregarded (21). Without complementary information about the reasons for missing games in our sample, it is impossible to ground the reported association to prior empirical research.

On the other hand, the negative and significant correlation found in the ice hockey athletes' group between Neurotracker average performance and total penalty minutes could be the most interesting result of the present study, where athletes with lower scores were more likely to collect higher amounts of penalty minutes. Poltavski and Biberdorf (16) suggested that university hockey athletes with low simple reaction time, but poor level of information processing and decision-making may react too quickly leading to bad decision-making which then leads to penalty (16). This could in part explain the highlighted finding of our study.

The remaining results of this study somewhat align with Tétreault et al. (1), where no association between Neurotracker performance and hockey game-related statistics was reported (17). There is a limited pool of literature on talent identification through Neurotracker performance, however, in a professional basketball context Mangine et al. (15) showed that NBA players with higher Neurotracker performance had a greater assist/turnover ratio (15), suggesting that athletes with greater abilities to process information and execute actions that integrate several stimuli are more likely to make better decisions, resulting in more positive plays. In our study, participants were highly trained athletes but were not professional athletes, and this could in part explain the fact that we did not find such associations. As such, professional athletes have different physiological characteristics, tactical components and higher performance standards (22). Our findings could also be explained by the nature of the selected player statistics, which may not be representative of perceptual-cognitive abilities measured by the Neurotracker. Advanced hockey statistics like turnovers (takeaways and giveaways) could be interesting to investigate in the future. These statistics could be more related to hockey players' ability to perceive and react to different stimuli on the ice, which would show demands similar to the Neurotracker.

### 4.1 Study limitations

Some study limitations such as small sample size, especially in the case of female volleyball, varsity sport representation, and data being non-normally distributed are worth mentioning., Furthermore, no sex-based analyses were conducted amongst participants, even though it has been previously reported that biological sex differences in Neurotracker performance exist (23, 24). This sub analysis was not warranted due to small sample size and to the different cognitive demands of ice hockey and volleyball, as previously reported by Acquin et al. (23). Finally, the significant negative relationship between games played and baseline Neurotracker performance remains to be studied and understood, as a significant correlation does not imply causation and could rather be caused by sample size or choice of statistics.

Despite these limitations, our study still contributes to the existing body of literature on the Neurotracker in athletes. In addition, to our knowledge, this study is the first to explore the correlation between Neurotracker performance and volleyball performances.

### 4.2 Future research

Further research should consider advanced statistics to examine the potential of the Neurotracker as a correlate of performance in a university varsity sports context. It would be interesting to explore the association between Neurotracker performance and more advanced sport statistics that may be more specific to perceptual-cognitive skills, with a greater pool of university varsity athletes of both biological sexes.

## 5 Conclusion

In conclusion, Neurotracker performance seems to be negatively correlated to cumulative penalty minutes in ice hockey players. Remaining basic sport statistics were not found to be linked to Neurotracker performance. Our study showed no significant associations between Neurotracker performance and sports statistics that could justify the use of the Neurotracker as a talent or performance identification tool in a university varsity hockey and volleyball context, through basic and readily available sport statistics. Should Neurotracker deem to correlate to advanced sports statistics in future studies, the role of repeated exposure to the training paradigm to enhance overall sport performance, as reported by sports statistics, could highlight its use as a longitudinal training tool in athletes.

## Data Availability

The datasets presented in this article are not readily available because data will be made available upon reasonable request. Requests to access the datasets should be directed to laurie-ann.corbin-berrigan@uqtr.ca.
